# Surgical aortic valve replacement in elderly patients: effects on physical performance, cognitive function and health-related quality of life

**DOI:** 10.1007/s40520-021-01969-x

**Published:** 2021-08-26

**Authors:** Marina De Rui, Vincenzo Tarzia, Mattia Mazzochin, Anna Bertocco, Chiara Ceolin, Caterina Trevisan, Chiara Tessari, Chiara Cavalli, Antonio Piperata, Alessandra Coin, Gino Gerosa, Giuseppe Sergi

**Affiliations:** 1 Department of Medicine-DIMED, Geriatrics Division, Clinica Geriatrica, University of Padova, via Giustiniani 2, 35128 Padova, Italy; 2 Department of Cardiac, Thoracic and Vascular Sciences, and Public Health, Cardiac Surgery Unit, University of Padova, Padova, Italy

**Keywords:** Aortic valve stenosis, Older adults, Nutritional status, Short physical performance battery, Mood, Quality of life

## Abstract

**Background:**

Surgical aortic valve replacement (SAVR) is still the gold standard for treating aortic valve stenosis (AVS). Its effectiveness has been extensively examined in terms of perioperative mortality, but its impact on overall health has received much less attention.

**Aims:**

To assess the physical performance, cognitive status, and health-related quality of life of elderly patients undergoing SAVR, in the short, medium and long term.

**Methods:**

This single-center prospective study enrolled patients aged > 70 years who underwent isolated SAVR for severe AVS. Data were collected on each participant’s clinical status, physical performance, cognitive status, mood, and health-related quality of life. This multidimensional geriatric assessment was performed before surgery (T0), and again at 45 days (T1), 3 months (T2), 6 months (T3), and 12 months (T4) post-surgery. Baseline (T0) and follow-up (T2-T4) data were compared separately for patients grouped by gender using paired t-tests.

**Results:**

Data from a total of 35 patients were analyzed. Compared with the baseline (T0), nutritional status worsened at T1, then gradually improved through to T4. Physical performance, mood, and health-related quality of life improved significantly after surgery. Cognitive function showed no change through to T3, but then deteriorated at T4.

**Conclusions:**

Our results show that SAVR in patients over 70 years of age has a positive impact on nutrition, mood, and health-related quality of life. Cognitive function was not negatively affected in the short and medium term, although it deteriorated in the long term. SAVR also had a positive impact on the physical performance of our sample.

## Introduction

Aortic valve stenosis (AVS) is a common heart valve disease with increasing prevalence due to the aging of the population. Around 13.2% of patients with AVS are over 75 years of age [1, 2]. Patients diagnosed with AVS remain asymptomatic for decades, while symptoms of dyspnea, angina and syncope develop from the 6th to 8th decade of life [3]. It is recognized that the prognosis is very poor once these symptoms occur, the mean survival being 23 ± 5 months. Early aortic valve replacement (AVR) should therefore be highly recommended in all symptomatic patients [4]. Surgical aortic valve replacement (SAVR) is a well-established procedure that is performed via a full median sternotomy under general anesthesia with the support of a cardiopulmonary bypass (CPB) and aortic cross-clamping (ACC). Although the long-term outcomes in terms of event-free survival and quality of life are excellent (for almost all patient risk profiles) [5], novel techniques have nonetheless been adopted in recent decades. These include minimally invasive approaches to contain postoperative morbidity and enhance patient satisfaction, and transcatheter aortic valve replacement (TAVI) to avoid CPB and ACC (which are known to be associated with increased rates of morbidity and mortality) [6]. Despite its advantages, widespread use of TAVI is still a matter of debate, largely because of uncertain long-term outcomes [7]. In fact, transcatheter approaches are only recommended in cases where surgery is considered high-risk or unsuitable, and is always subject to a multidisciplinary, preoperative patient assessment. There is still no clear definition of a high-risk patient, and meanwhile it rests on a multidimensional assessment that includes age, comorbidities, and other characteristics covered by the concept of frailty [8]. Physical performance and frailty are predictors of disability and mortality in elderly patients [9], including in the context of cardiac surgery [10]. The relevance of physical performance in the elderly is supported by the finding that adding the functional parameter gait speed to the Society of Thoracic Surgery (STS) risk score of patients undergoing cardiac surgery resulted in a two- to threefold improvement in its ability to predict in-hospital morbidity and mortality [11].

Several studies have examined the effectiveness of SAVR in older patients in terms of postoperative mortality [8, 9], but its impact on physical and mental performance, and overall health has not been widely investigated.

The aim of the present study, therefore, was to assess the physical performance, cognition, health-related quality of life, and frailty of elderly patients undergoing SAVR, in the early postoperative period and over the following 12 months.

## Subjects and methods

This retrospective observational study was designed and conducted jointly by the Geriatric Section of the Department of Medicine and the Cardiac Surgery Unit of the University of Padova. The study was conducted from February 2017 to August 2019.

Caucasian subjects > 70 years of age with severe symptomatic AVS and indicated for isolated SAVR were recruited for the study. Recommendation for SAVR was made by a Heart Team following joint discussion.

The study was designed in accordance with the Helsinki Declaration. All participants were made fully aware of the nature, purpose, procedures and risks of the study, and gave their informed consent. The study was approved by the Local Ethical Committee (Prot. N.0022928).

**Fig. 1:**
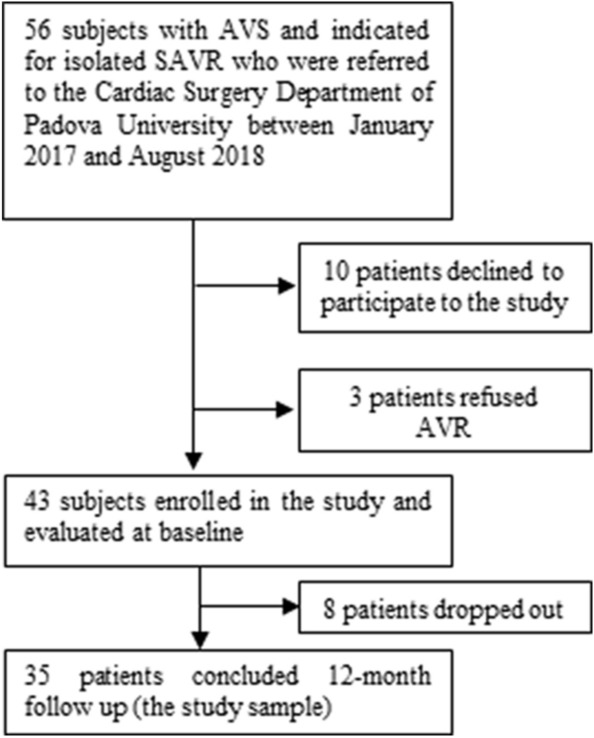
Flowchart of patient selection process. *AVS* aortic valve stenosis; *AVR* aortic valve replacement

Figure 1 illustrates the patient selection process. Of the 43 patients initially enrolled, 35 completed the 12-month follow-up and were included in the present analysis. Of the other eight, seven were unwilling to continue to follow-up, and one patient died in the meantime.

All patients underwent SAVR via a sternotomy under general anesthesia, with CPB and ACC, and all attended a period of cardiac rehabilitation after discharge from hospital.

The patients were assessed by trained medical personnel before surgery (T0), and then again at 45 days, and at 3, 6 and 12 months after SAVR (T1, T2, T3 and T4, respectively).

At each time point, patients underwent a comprehensive clinical examination comprising:

1. Anthropometric measurements: body weight was measured to the nearest 0.1 kg with standard scales, and height to the nearest 0.1 cm with a stadiometer (Seca; Germany) with subjects wearing light clothing and no shoes. Their BMI was calculated as their weight in kilograms divided by the square of their height in meters.
2. Comorbidities and disease severity indicators: these were assessed using the Cumulative Illness Rating Scale (CIRS) [12], which classifies comorbidities into 13 organ systems, and grades each condition from 0 (no problem) to 4 (severely incapacitating or life-threatening). The comorbidity index (CIRS-CI) is calculated on the basis of the number of conditions graded ≥ 3. The severity index (CIRS-SI) is the mean of the severity scores for each of the 13 organ systems.
3. Functional assessment based on the Activities of Daily Living (ADL) [13], and the Instrumental Activities of Daily Living (IADL) [14] indices; given the different traditional roles of men and women in Italian families, 3 items (preparing meals, doing housework, and doing laundry) were not applied to the men. To ensure comparability of values, IADL scores were calculated as percentages of the maximum value.
4. Nutritional status was assessed by the 18-item Mini Nutritional Assessment (MNA) tool [15]. A total MNA score ≥ 23.5 indicates individuals with good nutritional status, scores between 17 and 23.5 those at nutritional risk, and < 17 those with protein-calorie malnutrition.
5. Physical performance was measured with the following tools:
  I. the Short Physical Performance Battery (SPPB) [16], which consists of 3 objective physical function tests, i.e., 4-m gait speed, repeated chair stands, and standing balance in increasingly challenging positions; each test was scored from 0 (worst) to 4 (best), with the scores from all three tests combined to obtain a composite score of 0–12, where higher scores reflect better physical function;
  II. the 6-min walking test (6-MWT) [17]: participants were asked to walk at their usual pace for 6 min, and the distance they covered was recorded in meters; a difference of 54 m between tests at different time points was taken to indicate a clinically significant variation;
  III. handgrip strength was measured on the dominant side with a DynEx electronic hand dynamometer; 3 measurements were taken with a 1-min rest between trials, and the highest was used in our analyses; handgrip endurance was measured by asking subjects to maintain 50% of maximum voluntary contraction for as long as they could, and the time was recorded in seconds with a stop watch [18].
6. Cognitive status was measured via the Montreal Cognitive Assessment (MoCA) [19], which covers multiple cognitive domains. Scores of ≥ 26/30 are considered normal.
7. Affective status was measured with the Geriatric Depression Scale (GDS) [20], a 30-item self-reporting tool validated for use with the elderly. Scores < 10 indicate no depression, scores of 11–16 indicate mild-to-moderate depression, and scores > 17 indicate severe depression.
8. Health-related quality of life was assessed with the short-form 36-item health survey (SF-36) [21], which comprises 36 multiple-choice questions sorted into 8 subscales that describe overall health status. These subscales are: physical functioning (PF); role limitations due to physical problems (PR); bodily pain (BP); general health perception (GH); vitality (VI); social functioning (SR); role limitations due to emotional problems (ER); and general mental health (MH). Low numerical scores reflect a perception of poor health, loss of function, and presence of pain. The SF-36 items were coded and scored as explained in the SF-36 manual; a score > 50 for each item was considered indicative of an “adequate” perceived health status [22].

### Statistical analysis

The data for the analysis consisted of all the measurements taken at the baseline and at the follow-up assessments. Participants’ characteristics were summarized as means ± standard deviations for continuous variables, and counts and percentages for categorical variables. Normal distribution of the continuous variables was checked using the Shapiro–Wilk test. The non-parametric Mann–Whitney test was used to check differences between the medians of the SPPB scores.

Baseline characteristics of patients grouped by gender were compared using independent *t* tests, Chi-square tests, or Fisher’s exact test, as appropriate. Paired *t* tests were used for within-group comparisons of the baseline and follow-up data.

All analyses were performed in SPSS for Windows 21.0 (IBM Corp, Armonk, NY). All statistical tests were two-tailed, and statistical significance was set at a *p* value of < 0.05.

## Results

### Baseline characteristics of the sample

Table 1 shows the general characteristics of the sample grouped by gender.

**Table 1:** General characteristics of the subjects at baseline by gender

	Women (*n* = 12)	Men (*n* = 23)	p value (women vs men)
Age (years)	74.2 ± 3.2	76.7 ± 3.8	n.s
Echocardiographic parameters			
Indexed aortic valve area (cm^2^/m^2^)	0.48 ± 0.15	0.84 ± 1.02	n.s
Peak transaortic pressure gradient (mmHg)	83.4 ± 33.7	70.19 ± 21.03	n.s
Mean transaortic pressure gradient (mmHg)	49.8 ± 20.2	45.57 ± 11.53	n.s
Left ventricular ejection fraction (%)	60.0 ± 6.9	53.3 ± 8.1	n.s
Multidimensional assessment			
Comorbidities			
CIRS-CI score	4.0 ± 1.5	3.7 ± 1.2	n.s
CIRS-SI score	1.9 ± 0.2	1.8 ± 0.2	n.s
Nutritional assessment			
BMI (kg/m^2^)	30.1 ± 5.5	28.6 ± 3.0	n.s
MNA score	23.8 ± 1.7	25.1 ± 2.0	n.s
MNA 17–23.5 (% of subjects)	50.0	21.7	n.s
MNA < 17 (% of subjects)	0	0	–
Functional status			
Independent in ADL	5.8 ± 0.4	5.9 ± 0.3	n.s
Independent in IADL %	94.8 ± 8.3	95.6 ± 17.0	n.s
Physical performance			
SPPB total score [median (IQR)]	10 (8–11)	10 (9–12)	n.s
Gait speed (m/s)	1.21 ± 0.28	1.03 ± 0.30	n.s
6-MWT (m)	280.9 ± 130.6	400.6 ± 85.6	0.003
Handgrip maximal strength (kg)	21.9 ± 6.4	38.2 ± 10.2	< 0.0001
Handgrip endurance (sec)	78.3 ± 67.2	79.7 ± 36.8	n.s
Cognitive status and mood			
MoCA score	21.3 ± 4.2	24.9 ± 2.3	0.027
GDS score	13.5 ± 4.1	11.9 ± 3.5	n.s
GDS 11–16 (% of subjects)	41.6	30.4	n.s
GDS > 16 (% of subjects)	24.9	8.3	n.s

*CIRS* Cumulative Illness Rating Scale (*CI* Comorbidity Index, *SI* Severity Index), *BMI* body mass index, *MNA* mini nutritional assessment, *ADL* activities of daily living, *IADL* instrumental activities of daily living, *SPPB* short physical performance battery, *IQR* interquartile range, 6-MWT 6-min walking test, *MoCA* montreal cognitive assessment, *GDS* Geriatric Depression Scale

The majority of subjects were in New York Heart Association (NYHA) class 2, while approximately one in four were in NYHA class 3.

The multidimensional assessments showed that our subjects had an average of four comorbidities, with no differences between genders. According to the results of the MNA, 50% of the women and 21.7% of the men were at risk of malnutrition.

The ADL and IADL scores were similar for both genders. Regarding physical performance, the women covered a significantly shorter distance than the men in the 6-MWT, and had lower handgrip strength scores.

The MoCA scores of the women were significantly lower than those of the men: 88.9% of the women, but only 54.5% of the men had MoCA scores < 26/30. GDS scores showed that 41.6% of the women and 30.4% of the men had mild-to-moderate depression, and 24.9% of the women and 8.3% of the men had severe depression.

Regarding health-related quality of life, most of the patients scored < 50 on all the SF-36 items, indicating an unsatisfactory quality of life.

### Comparisons between baseline and 45-day follow-up

As Table 2 shows, there was a significant deterioration in patients’ nutritional status at 45 days after surgery compared with baseline. Their BMI and their MNA scores decreased significantly, and a higher number of patients were at risk of malnutrition. There were no differences in cognitive function or mood (MoCA, GDS scores), but the percentage of patients with a GDS score ≥ 11 decreased significantly (from 65.6 to 45.0%, *p* = 0.01). Regarding functional status, there were no differences in ADL scores, but the IADL scores, which indicate the patient’s level of independence, decreased significantly (from 95.3% ± 14.4 to 84.7% ± 24.9; *p* = 0.014). Physical performance, as measured by gait speed and the 6-MWT, did not differ significantly. Among the SPPB items, standing balance deteriorated significantly compared with baseline (*p* = 0.05). The proportion of patients with total SPPB scores ≤ 8 increased from 22.9 to 35.5% (*p* = 0.035). Handgrip maximum strength also declined significantly. There were no significant differences in SF-36 scores, which represent health-related quality of life.

**Table 2:** General characteristics of the sample (*n* = 35) at baseline and over the follow-up period

	Baseline (T0)	45-day FU (T1)	3-month FU (T2)	6-month FU (T3)	12-month FU (T4)	*p* value (T0 vs T1)	*p* value (T1 vs T2)	*p* value (T0 vs T2)	*p* value (T0 vs T3)
Nutritional assessment									
BMI (Kg/m^2^)	29.1 ± 4.1	27.3 ± 3.7	28.1 ± 4.4	28.4 ± 4.1	29.1 ± 4.4	< 0.0001	0.013	n.s	n.s
MNA score	24.6 ± 2.0	22.6 ± 2.9	25.0 ± 2.3	25.9 ± 2.6	25.8 ± 1.6	0.003	< 0.0001	n.s	0.023
MNA score 23.5–17 (%)	31.4	51.4	17.1	14.3	11.4	0.006	< 0.0001	0,02	0.003
MNA score < 17 (%)	0.0	2.9	0.0	0.0	0.0	0.05	0.05	n.s	n.s
Functional status									
Independent in ADL	5.9 ± 0.3	5.7 ± 0.6	5.8 ± 0.5	5.8 ± 0.6	5.8 ± 0.6	n.s	n.s	n.s	n.s
Independent in IADL %	95.3 ± 14.4	84.7 ± 24.9	90.2 ± 19.2	96.5 ± 17.1	93.4 ± 21.3	0.014	n.s	n.s	n.s
Physical performance									
SPPB total score [median (IQR)]	11 (8–12)	12 (9–12)	11 (9–12)	12 (11–12)	12 (11–12)	n.s	n.s	n.s	0.028
Subjects with SPPB total score ≤ 8 (%)	22.9	35.5	18.5	9.1	22.9	0.035	0.015	0.024	0.01
Gait speed (m/s)	1.08 ± 0.30	1.17 ± 0.30	0.97 ± 0.32	1.02 ± 0.29	1.16 ± 0.47	n.s	n.s	n.s	n.s
6-MWT (m)	361.8 ± 11.5	361.0 ± 12.4	383.0 ± 19.4	368.3 ± 125.4	362.3 ± 130.7	n.s	0.048	n.s	n.s
Handgrip maximal strength (kg)	32.6 ± 11.9	28.6 ± 10.7	31.4 ± 12.6	32.4 ± 12.1	30.2 ± 11.8	0.008	n.s	n.s	n.s
Handgrip endurance (sec)	79.2 ± 48.3	67.5 ± 38.1	73.9 ± 38.3	67.3 ± 25.9	69.8 ± 31.8	n.s	n.s	n.s	n.s
Cognitive status and mood									
MoCA score	23.2 ± 3.7	22.6 ± 5.2	22.7 ± 3.6	22.6 ± 5.0	20.1 ± 5.1	n.s	n.s	n.s	n.s
MoCA score < 26/30 (%)	70.0	60.0	71.4	61.1	88.5	n.s	n.s	n.s	n.s
GDS score	12.6 ± 3.8	10.5 ± 4.2	10.2 ± 6.6	7.2 ± 6.3	4.6 ± 5.2	n.s	n.s	n.s	n.s
GDS score ≥ 11 (%)	65.6	45.0	48.8	26.4	7.6	0.01	n.s	0.006	< 0.0001

*FU* follow-up, *BMI* body mass index, *MNA* mini nutritional assessment, *moca* montreal cognitive assessment, *GDS* Geriatric Depression Scale, *ADL* activities of daily living, *IADL* instrumental activities of daily living, SPPB short physical performance battery, *IQR* interquartile range, 6-*MWT* 6-min walking test

### *Variations in the multidimensional assessment domains at 3, 6 and 12 months after SAVR (Table **2**)*

#### Nutritional assessment

While BMI was significantly lower 3 months after SAVR than at baseline, it no longer differed from baseline at the 6- and 12-month follow-ups. MNA scores at the 3-month follow-up did not differ significantly from baseline, and were significantly higher than at the 45-day follow-up. At 6 months, MNA scores were significantly higher than at baseline (rising from 24.6 ± 2.0 to 25.9 ± 2.6, *p* = 0.023), and a smaller percentage of patients had scores in the range of 17–23.5 (down from 31.4 to 14.3%, *p* = 0.001). Twelve months after SAVR, MNA scores remained the same as after 6 months, and an even smaller proportion of patients had MNA scores in the range of 23.5–17 (11.4%), while none had scores indicating malnutrition (MNA scores < 17).

#### Physical performance

ADL and IADL scores at the 3-, 6- and 12-month follow-ups were not significantly different from baseline.

The only SPPB score that differed significantly from baseline at the 3-month follow-up was for repeated chair stands, which showed patients improving significantly (*p* = 0.05). At 6 and 12 months, the 4-m gait speed, repeated chair stands, and total SPPB scores all improved significantly compared with the baseline. The percentage of patients with a total SPPB score ≤ 8 dropped significantly at the 3-month follow-up (from 22.9 to 18.5%, *p* = 0.024), and at 6 months, but at the 12-month follow-up it did not differ significantly from the baseline. There were no differences in the 6-MWT, gait speed, or handgrip maximum strength and endurance at the 3-, 6-, and 12-month follow-up assessments.

#### Cognitive function and mood

There were no changes in MoCA scores at the 3- and 6-month follow-ups compared with the baseline and 45-day follow-up. At 12 months, the patients’ MoCA scores decreased significantly compared to the baseline (from 23.2 ± 3.7 to 20.1 ± 5.1, *p* = 0.026), and the proportion of patients scoring < 26/30 rose from 70 to 88.5% (a statistically non-significant difference).

At the 3- and 6-month follow-ups, the patients’ mean GDS scores did not differ significantly from the baseline, but by 12 months after SAVR their scores had improved significantly (from 12.6 ± 3.8 at baseline to 4.6 ± 5.2, *p* < 0.0001). The percentage of patients with GDS scores ≥ 11 was significantly lower at 3 months than at baseline (dropping from 65.6 to 48.8%, *p* = 0.006), and decreased further at 6 months (to 26.4%), and at 12 months (to 7.6%).

#### Health-related quality of life

**Fig. 2:**
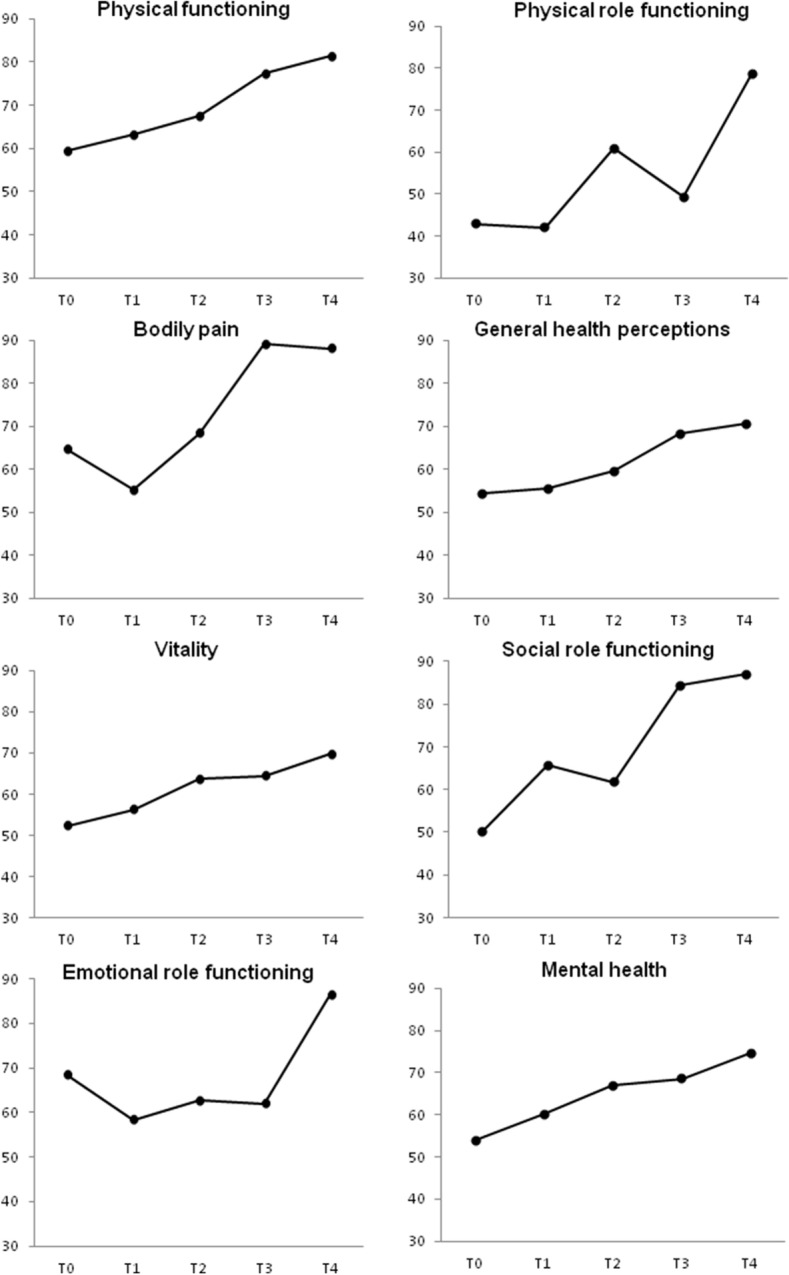
Mean scores of the study population for SF-36 items at baseline (T0), and at 45 days (T1), 3 months (T2), 6 months (T3), and 12 months (T4) after surgery. *Significant variations:* physical functioning: T0 vs T4 (p = 0.01); role limitations due to physical problems: T1 vs T2 (p = 0.02); T3 vs T4 (*p* < 0.0001); bodily pain: T1 vs T2 (*p* = 0.01); T0 vs T4 (*p* = 0.011); vitality: T1 vs T2 (*p* = 0.02); T3 vs T4 (*p* = 0.03); T1 vs T4 (*p* = 0.001)

**Fig. 3:**
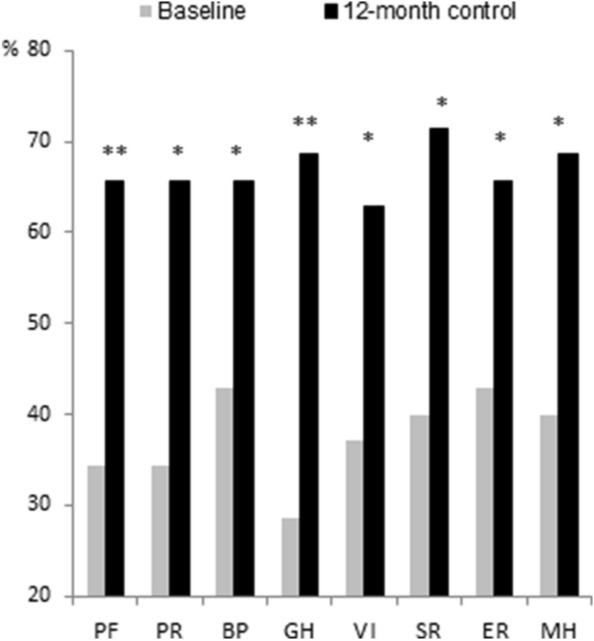
Comparison of the percentages of subjects with SF-36 scores > 50 at baseline and at the 12-month follow-up. *PF*: physical functioning; *PR* role limitations due to physical problems; *BP* bodily pain; *GH* general health perceptions; *VI* vitality; *SR* social functioning; *ER* role limitations due to emotional problems; *MH* mental health. *p < 0.05; **p < 0.01

As Fig. 2 shows, compared with the baseline, there was an improvement 12 months after SAVR in the scores reflecting physical functioning (up from 59.5 ± 23.9 to 81.3 ± 20.9, *p* = 0.011), and bodily pain (up from 64.7 ± 23.5 to 88.2 ± 21.1, *p* = 0.011). Moreover, the number of patients with satisfactory SF-36 scores on all items was significantly higher at 12 months than at baseline (Fig. 3).

## Discussion

The present study explored the short-, medium- and long-term impact of SAVR on the functional and neuropsychological status of elderly patients.

As our focus here was on surgical AVR, the patients comprising our sample were younger than those who usually undergo TAVI. In clinical practice, in fact, aortic valve replacement is usually indicated in patients in advanced stages of the disease and in older age groups, and where SAVR is considered riskier than TAVI. Nevertheless, earlier intervention may limit the negative consequences of a prolonged history of aortic valve stenosis; on the other hand, SAVR is more invasive than TAVI, and could therefore have a greater traumatic impact on the overall health status of elderly patients.

In the medium term, there was an improvement in patients’ nutritional, neuropsychological and functional statuses, and perceived health-related quality of life, but no change in their muscle strength. At the 12-month follow-up, their nutritional status, physical functioning and perceived health-related quality of life had significantly improved, while their cognitive function had slightly worsened.

Regarding nutritional status, 28.6% of patients were at risk of malnutrition. This finding is in line with Goldfarb et al. [23], who found 32.8% of patients aged ≥ 70 years undergoing SAVR at risk of malnutrition. In patients undergoing general surgery, malnutrition is associated with delayed wound healing, postoperative complications, prolonged hospital stays, readmissions, and death [24]. In the case of SAVR, Goldfarb et al. [23] found that the crude 1-year risk of mortality was 3 times higher in malnourished patients than in those with a normal nutritional status.

Surgical intervention had a marked impact on the nutritional status of our sample at the 45-day follow-up, when body weight was down by 2.6 ± 2.0 kg, and the proportion of patients at risk for malnutrition had almost doubled. The weight loss is probably due to a lower calorie intake and to hypermetabolism, as also demonstrated by Sallè et al. [25]. This deterioration in the nutritional status of the patients in our sample was reversible, and by 3 months post-surgery their BMI and MNA scores, and the percentage of those at risk of malnutrition were no different from the baseline. By 6 months after surgery, MNA scores had increased significantly, fewer patients were at risk of malnutrition, and none had scores to indicate malnutrition. This improvement was maintained at the 12-month follow-up, suggesting that SAVR has a positive impact on nutritional status in the elderly.

Judging by their SPPB scores, the physical performance of our patients at baseline was satisfactory. In fact, baseline SPPB scores were very high, with only 9.5% of patients having a total score ≤ 8, the cutoff commonly used to diagnose sarcopenia [26]. Immediately after surgery, the proportion of patients with SPPB scores ≤ 8 increased significantly to 35.5%, but 6 and 12 months later it dropped back to about 9% (similar to the prevalence at baseline). The patients’ scores for repeated chair stands and 4-m gait speed were also better 6 months after than before surgery. This functional improvement in the medium term after SAVR is particularly noteworthy because the SPPB is considered to be a highly sensitive indicator of overall health status [27]. As this test battery also has a strong, independent ability to predict mortality, morbidity and hospitalization in older adults [28], an improvement in SPPB score may in the long term translate into a greater ability to respond to future stressors. As far as we know, only Kotajarvi et al. [29] have as yet examined the effect of SAVR on physical performance. In a sample of 103 elderly patients, they found that those patients who showed a greater improvement 3 months after surgery were those whose performance at baseline was lower. This means that preoperative assessments should not a priori exclude patients with low physical performance, since they might benefit the most from SAVR.

The transient worsening in standing balance observed 45 days after surgery may be an early consequence of deconditioning and muscle atrophy due to surgery and bed rest, which particularly affect the anti-gravity muscle groups that are very important for posture [30].

The results of the 6-MWT did not vary to a statistically significant degree from baseline to the 6- and 12-month follow-ups, although 20% of our patients walked > 54 m further at their 12-month follow-up test (commonly considered a clinically significant improvement). The fact that only 20% of patients made this improvement is probably due to the good mean performance of the sample at baseline. In fact, the subgroup of patients whose 6-MWT results did not improve during the study period had covered a significantly longer distance at the baseline assessment than the subgroup of patients who made an improvement after SAVR (392.6 ± 89.7 m vs 293.3 ± 146.1 m, respectively; *p* = 0.03).

Regarding muscle strength, our sample’s handgrip strength at baseline was comparable to that of the age-matched population [31]. At the first follow-up, their handgrip strength had significantly deteriorated as a consequence of the surgery and bed rest, but at the 3-, 6- and 12-month follow-up assessments, it was again the same as at baseline. This means that the improvement in functional performance after SAVR as shown by the SPPB was not related to any improvement in muscle strength, but instead reflected better overall health.

Regarding cognitive status, the MoCA identified impaired cognition in 80% of our sample at baseline. Since SAVR requires general anesthesia, our preliminary hypothesis was that surgery and hospitalization would negatively affect our elderly patients’ cognitive performance. However, we found no significant worsening of their cognitive function in the short or medium term after surgery. In the longer term (at the 12-month follow-up), the patients’ MoCA scores were significantly lower than at baseline, and there was a parallel increase in the number of patients with MoCA scores below 26/30 (though the difference was not statistically significant). Given that previous publications have not found SAVR to affect cognitive function [32], we interpret this decline as a sign of latent cognitive impairment at baseline evolving over time.

When we examined our patients’ mood, their baseline mean GDS scores suggested mild-to-moderate depression, while the scores of 18% of patients indicated severe depression. During the follow-up, there was a significant improvement in these scores and a consequent increase in the proportion of patients without depression. This may be due to an improvement in patients’ perceived health-related quality of life, as also indicated by the trend in our sample’s SF-36 scores. Depression is a recognized risk factor for adverse outcomes in cardiovascular disease. Among 1,035 older adults undergoing SAVR, Drudi et al. [33] found depression at baseline to be associated with mortality at one month (OR 2.2) and at 12 months (OR 1.52), while depression persisting 6 months after the procedure was associated with a threefold higher mortality rate at 12 months (OR 2.98). The drop in the percentage of depressed patients in our sample over the follow-up also seems to suggest that the cognitive decline seen 12 months after SAVR was not due to a form of masked depression.

When our patients judged their health-related quality of life (QoL) at baseline, the majority scored < 50 in four of the SF-36 items. Immediately after surgery, their perceived QoL deteriorated in nearly all domains, but gradually improved at subsequent follow-up assessments, and was generally better at 12 months than at baseline, in agreement with Shan et al. [34]. A year after their surgical procedure, most patients reported an adequate QoL for all SF-36 items. Postoperative health-related QoL is a primary goal for elderly people, and an important factor in many patients’ decision to undergo surgery or not.

The present study has some limitations. First, the sample was small. This was due partly to our strict inclusion and exclusion criteria, and partly to the number of drop-outs. Our small sample size could have underestimated potentially significant benefits of SAVR.

Second, the low mortality rate (only one patient died) was naturally a positive outcome, but it meant that we were unable to explore any associations between baseline performance and mortality. Third, all patients underwent median sternotomy, so we could not draw any comparisons with patients undergoing other, less invasive, procedures, which might differently impact on the functional status of elderly subjects.

## Conclusions

Our data show that SAVR had no negative effects on the nutritional status of a very selected sample of young elderly patients. If anything, there seems to be a benefit, as demonstrated by the improvement in our patients’ MNA scores, and the gradual reduction in the proportion of those at risk of malnutrition at the 6- and 12-month follow-ups. A slight (and probably unrelated) deterioration in cognitive function was seen in the long term, but our patients’ physical performance improved after SAVR. Their total SPPB scores were significantly higher at the long-term follow-up, although there were no changes in gait speed or handgrip strength (considered to be indicators of good overall health). The patients showing the greatest benefit from SAVR were those performing the worst at baseline. A significant improvement in mood was evident at the long-term follow-up, when all patients also reported having a better health-related quality of life than at baseline, particularly with regard to physical functioning and bodily pain. Further investigation should be performed to confirm our results in a larger group of elderly patients.

## Data Availability

The datasets generated and/or analyzed during the current study are available from the corresponding author upon reasonable request.
